# Interlamellar Organization of Phase Separated Domains in Multi-Component Lipid Multilayers: Energetic Considerations

**DOI:** 10.3390/ijms14023824

**Published:** 2013-02-08

**Authors:** Lobat Tayebi, Atul N. Parikh, Daryoosh Vashaee

**Affiliations:** 1 Helmerich Advanced Technology Research Center, School of Material Science and Engineering, Oklahoma State University, Tulsa, OK 74106, USA; 2 School of Chemical Engineering, Oklahoma State University, Stillwater, OK 74078, USA; 3 Department of Applied Science, University of California, Davis, CA 95616, USA; 4 Department of Biomedical Engineering, University of California, Davis, CA 95616, USA; E-Mail: anparikh@ucdavis.edu; 5 Department of Chemical Engineering & Materials Science, University of California, Davis, CA 95616, USA; 6 Helmerich Advanced Technology Research Center, School of Electrical and Computer Engineering, Oklahoma State University, Tulsa, OK 74106, USA

**Keywords:** lipid multilayers, phase separation, aligned domains, energetic considerations, lipid rafts

## Abstract

A recent experimental study [1] has demonstrated the alignment of phase separated domains across hundreds of bilayer units in multicomponent stacked lipid bilayers. The origin of this alignment is the interlamellar coupling of laterally phase separated domains. Here, we develop a theoretical model that presents the energetics description of this phenomenon based on the minimization of the free energy of the system. Specifically, we use solution theory to estimate the competition between energy and entropy in different stacking configurations. The model furnishes an elemental phase diagram, which maps the domain distributions in terms of the strength of the intra- and inter-layer interactions and estimates the value of inter-layer coupling for complete alignment of domains in the stacks of five and ten bilayers. The area fraction occupied by co-existing phases was calculated for the system of the minimum free energy, which showed a good agreement with experimental observations.

## 1. Introduction

Phase separation and domain formation in multicomponent lipid bilayers containing immiscible liquid phases is a well-known phenomenon. In this regard, ternary lipid mixtures consisting of cholesterol (Chol), sphingomyelin (SM), and unsaturated phospholipids are of considerable interest as models for lipid rafts, which are thought to be relevant in a variety of cell-surface signaling in biological membranes [2–5].

These lipid mixtures, below their miscibility transition temperatures, readily phase-separate into two co-existing liquid phases: (1) liquid-ordered (L_o_) and (2) liquid-disordered (L_d_) [6–9]. L_o_ domains, or the so called lipid rafts, predominantly consist of Chol and SM. L_d_ domains are enriched in unsaturated phospholipid. Recently, we investigated these raft-forming ternary mixtures as stacked lipid bilayers (lipid multilayers), which led to the interesting observations of the long-range interlayer alignment of laterally phase separated L_o_ domains [1]. We found that the domains formed in each bilayer become aligned across several lamellae in a stacked lipid bilayer.

The demonstrated bio-epitaxial film is of considerable technological interest for biomimicry. For instance, thylakoid membranes of photosynthetic cyanobacteria [10] or plant chloroplasts [11], and electrocyte cells in electric eels [12] contain multilamellar membranous structures and their mimicry can be exploited in energy harvesting technologies. Potential applications of such membranes in photonics and sensing are also significant [13–15].

In the experimental investigation presented in [1] various compositions were examined including equi-molar SM and DOPC mixtures with different concentrations of cholesterol in the range of 10–40 mol% of the total solution [Cholx/SM1/DOPC1; *x* = 10%–40%]. The domain formation and their alignments were observed in all of such compositions. Their alignments were examined using a combination of X-ray diffraction, fluorescence confocal microscopy, and atomic force microscopy measurements. In this study, we also carried out additional experiments to validate the theoretical model, which is the main subject of the current paper.

**Figure 1:**
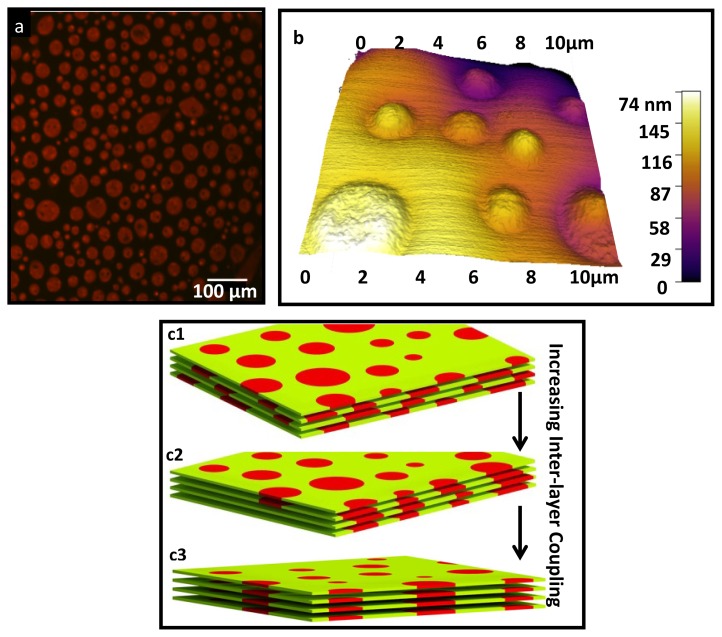
(**a**) Epiflorescence image of the top surface of a multi component lipid multilayers doped with 0.03 mol% of Rhodamine which partition preferebly with L_d_ domains. Scale bar is 100 μm; (**b**) Atomic force microscopy image of the multilayer cleraly shows the aligned domains; (**c1**,**c2**) Entropy favors the randome distribusion of the domains in different bilayers of the multilayer as shown in panel (**c1**); However, the inter-layer couling competes with entropy to align the domains. More coupling strengh lead to more complete alignmnet (**c2**,**c3**).

Examples of the formation of the aligned domains in lipid multilayer and their epifluorescence and atomic microscopy (AFM) images are shown in Figure 1a,b. As can be seen in  Figure 1c1–c3, entropically a multilayer system prefers a random distribution of the domains. Alignment of the domains requires the existence of a strong coupling between bilayers which can overcome the entropy. It was suggested that the origin of this coupling is the energy penalty associated with the tension between the different water networks at the vicinity of the different domains [1]. It was suggested that the origin of this coupling is the energy penalty associated with the tension between the different water networks at the vicinity of the different domains [1].

To analyze this coupling and estimate its value, here we investigated the competition of the energy and the entropy in multicomponent lipid multilayer systems with varying number of lamellae. We derived an elemental phase diagram that can explain the relation between the inter- and intra-layer coupling parameters. The phase diagram can predict the production of the multilayers with different domain distributions (different phases). It can further estimate the intra-layer coupling value for the complete alignment of the multilayers. The results are presented for the two cases of 5 and 10 number of lamellae. The model calculation can also predict the areal fraction of the multilayer surface adopted by different phases. Presenting an example of this calculation, we show that the theoretical result is in good agreement with the corresponding experimental data.

## 2. Results and Discussions

Although the approach to the equilibrium for the domain dynamics in multilamellar lipid membrane, specifically at low humidity, is slow, membrane multilamellae ultimately do reach to a point that no further changes are observed at experimental time scales (several days) [1]. Under these conditions of quasi-equilibration, applying energetic considerations becomes instructive, such as the model described below.

To model the experimental system of multicomponent lipid mulilayer, we begin by considering each bilayer as a consolidated unit. In the other words, we assume that the domains, formed via intralamellar phase separation, are pre-aligned across the two leaflets of each bimolecular lamellar sheet (monolayers). Exactly similar to the experimental report in reference [1], we consider the composition of Chol, SM and 1,2-dioleoyl-sn-glycero-3-phosphocholine (DOPC) as the unsaturated lipid. Next, considering nearest–neighbor intra-layer interaction between the species, which is positive (unfavorable) between DOPC and Chol and negative (favorable) between SM and Chol [2,16], we assume SM and Chol represents one component and model our system as a pseudo-binary mixture [17] with component 1: DOPC and component 2: SM/Chol. These two components undergo phase separation to make two phases L_d_: DOPC-rich and L_o_: SM/Chol-rich. To account for the small non-ideal compositional variation in each layer, a normal distribution with mean value equal to the molar ratio of the composition and a small standard deviation of 0.02 is assumed.

The total free energy, *F*, of the multilamellar membrane can be approximated using the mean-field description of the regular solution theory. Within this framework, (1) assuming the random mixing approximation of Bragg-Williams type for each layer [18,19], (2) introducing a coupling term for the interaction between bilayers, (3) adding the additional entropy term due to the ordering multiplicity of the multilayer, and finally (4) summing over all lamellae, we can write:

(1) $$F=\frac{A}{\Phi_{1}a_{1}+\Phi_{2}a_{2}}\sum_{j=Phase\;number}t_{j}(E_{j}-S_{j})$$

Where

(2) $$E_{j}=\sum_{i=1}^{N}\left(\chi \phi_{ij}(1-\phi_{ij})+\Lambda_{ij}{\left(\phi_{ij}-\phi_{i+1,j}\right)}^{2}\right)$$

(3) $$S_{j}=\sum_{i=1}^{N}\left(-\phi_{ij}\;\text{ln}\phi_{ij}-(1-\phi_{ij})\;\text{ln}(1-\phi_{ij})\right)+\text{ln}\frac{N!}{\prod_{\ell =1}^{k}n(\ell ,j)!}$$

**Figure 2:**
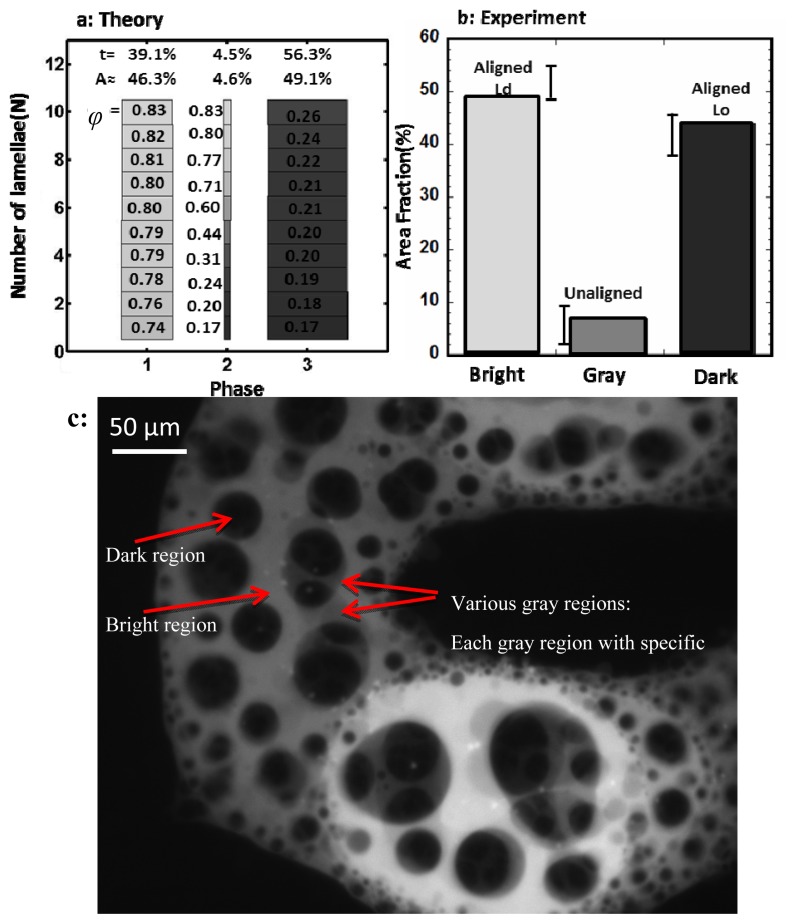
Simulation results for a 10-layer sample with *X* = 10%. (**a**) Three phases are predicted for χ = 2.3 and Λ = 0.7: phase 1: aligned L_d_, phase 2: unaligned, and phase 3: aligned L_o_. The local molar ratio of DOPC (ϕ) for each layer in each phase (ϕ*_ij_*) is shown in its corresponding box. The brighter the area, the higher the concentration of DOPC. The width of each box is proportional to the surface area of each domain. Each phase occupies “*t* mole%”, and “A area%” of the multilayer top surface; (**b**) Image analysis of several experiments in multilayer at *X* = 10% is summarized in this plot (variation bars are shown). The area occupied by the bright, gray, and dark domains (representing the fluorescent intensity in the experiment) is in good agreement with the area predicted for phases 1, 2 and 3 in plot a, respectively; (**c**) An example of a phase separated lipid multilayer: Gray regions indicate the unaligned domains. Each specific color of gray represent one “phase”. When there are various gray regions in the multilayer, the system is called multi-phase. When there is no gray region, the system is composed of only two phases (dark and bright). In this case we have a “two-phase” multilayer in which the domains are completely aligned. Note that this picture is chosen as the gray regions can be clearly seen so we can clarify the concept of the “phase” in our model. Usually, multilayers do not consist of such wide regions of gray area and very small area fraction is adopted by gray region as it is shown in Figure 1a.

Where “*F*” is the total free energy of the multilayer in units of *K_B_T* (thermal energy), and “*t*” is the mol fraction of each “phase”. Here, the term “phase” refers to a multilamellar region with specific local composition in each layer (e.g., Figure 2a) (Note that this definition of “phase” differs from L_o_ or L_d_ phase).

As it is described in Figure 2c, for this terminology, experimentally, different phases are distinguished by their corresponding intensities observed in the epifluorescence measurements. The intensity of each phase, as seen from the top surface, is the superposition of the intensity of all layers under that region. For instance, in the case of only two distinguishable intensities, dark and bright, the multilayer is represented in terms of two phases.

“Φ _1_” and “Φ _2_” are the input values of the model and indicate the average mole value of the first and second component in the media, respectively. In other words, they are the mean composition (molar ratio) of component 1 (DOPC) and component 2 (SM/Chol) in the primary total stock solution. “*a*_1_” and “*a*_2_” are the molecular surface area of each component. “A” represents the total top surface area of the multilamellar sample. “*E_j_*” and “*S_j_*” refer to the energy and entropy parts of the total free energy in each phase, respectively. “*N*” is the number of lamellae. “ϕ*_ij_*” is the local composition of one component (e.g., DOPC) in phase *j* and layer *i*. “χ” is the degree of non-ideality expressing the strength of the nearest-neighbor interaction in a given layer. For simplicity, we refer to it as intra-layer coupling parameter. We assume χ is similar for all the layers. “*Λ*” is the inter-layer coupling parameter, which is assumed to be constant (*i.e.*, *Λ_ij_* = *Λ*). Each phase *j* may consist of several repeating ϕ*_ij_*s, which can be grouped together (e.g., in the first phase in Figure 2a two *ϕ* = 0.80 make a group). “*k*” is the number of such groups in that phase (e.g., in the first phase in Figure 2a *k* = 2). “ ” is the number of members in each group (e.g., in Figure 2a, *n*(1,1) = 2 and *n*(2,1) = 2). In Equation 2, the first term corresponds to the nearest-neighbor interaction in each layer. The second term is the energy penalty due to the local compositional mismatch between the adjacent bilayers. In Equation 3, the first two terms are the solution entropy associated with each layer. The last term is due to the entropy increase by the additional multiplicity due to repeating *ϕ_ij_*s in each phase *j*. This term increases significantly with the number of lamellae. *a_1_* is taken 0.69 nm^2^ for all compositions [20]. *a*_2_ is different for different concentrations of Cholesterol. For *X* = 10%, 15%, 20%, 30% and 40%, *a*_2_ values are taken as 0.48, 0.43, 0.40, 0.40, 0.39 and 0.39 nm^2^, respectively [21]. To find the most stable ϕ*_ij_s* and the corresponding *t_j_s*, *F* is minimized with respect to all variables under the following constraints:

$$\sum_{j}t_{j}=1\;\;\;\text{and }\;\;\sum_{j}t_{j}\varphi_{ij}=\Phi_{i}.$$

Where “Φ*_i_*” is the average composition of one component (e.g., DOPC) in layer *i*.

To further clarify the concepts of “phase” and “ϕ*_ij_*” let us consider a multilayer which is observed from the top surface such as the one shown in Figure 2c. In fluorescence imaging, the intensity in each point of the top surface of the multilayer is the superposition of intensities of all layers on that point. As a result, since the fluorescent molecules partition with only one type of lipid, the different colors (grays with different intensities) that can be seen on top surface of the multilayer, represent different columnar series of local lipid composition of all layers, which we call them different “phases”. For example, if we could visually distinguish three distinct colors on top of the total area of one multilayer, we would have three phases of that multilayer. Using the above equations, we can also calculate the number of phases in each multilayer. For example, Figure 2a which shows the result of the calculation performed for a multilayer consisting of ten lamellae (*N* = 10), contains three phases. A phase difference less than the variance of compositional fluctuation is ignored in the model. Each phase is shown by a column with 10 layers (10 boxes). The composition of DOPC in each phase and each layer is calculated and written in the corresponding box. This local composition of one specific lipid component in one phase is defined as ϕ*_ij_* (*I* = layer number and *j =* phase number). Other than the values of all ϕ*_ij_* in the multilayer system, the “t mole %”, and consequently the “area fraction %” of the multilayer top surface for all distinct phases, is also included in the outcomes of the model. These values give us the chance to compare the results of our model with the experiment as we can experimentally measure the area fraction of distinct phases in different multilayers.

To compare and verify the theoretical results with the experiment, several samples with similar composition were prepared following the method presented in [1]. In brief, the stack of the lipid mixture was incubated for two days at 50–60 °C followed by decreasing the temperature to room condition (~22 °C). Imaging and Epifluorescence measurements were performed using an inverted epifluorescence microscope (Nikon TE200E, Technical Instruments, San Francisco, CA, USA) equipped with a high spatial resolution CCD camera. The image analyses for estimating the surface area of the bright, dark and gray regions were performed using NIH Image J. Figure 2b shows a good agreement between the theoretical predictions and the experimental data.

**Figure 3:**
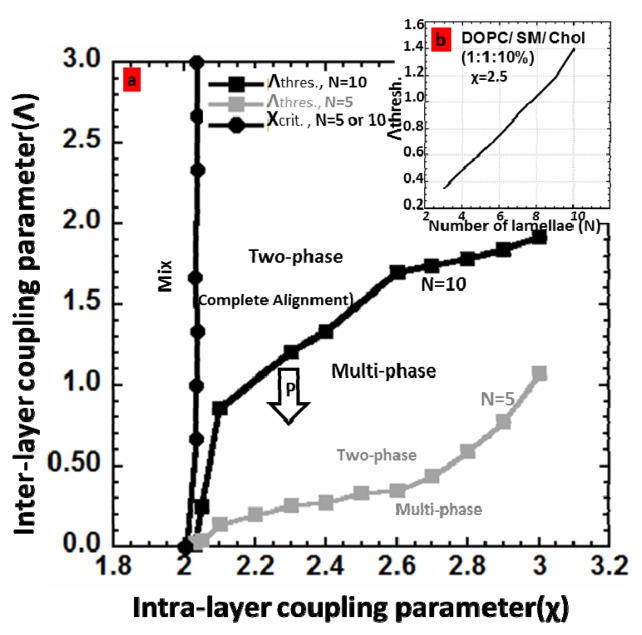
The phase diagram defining different regions of “mix”, “two phase”, and “multi phase” *versus* in-plane χ and cross-plane Λ coupling parameters. At and above *Λ_thres_* line, there are only two phases, “aligned L_d_” and “aligned L_o_”. Under *Λ_thres_* line, the system is multi-phase. Under χ*_crit_*, the system does not undergo phase-separation and will be fully mixed. c, An example of dependency of *Λ_thres_* to the number of lamellae for χ = 2.5.

Increasing *Λ* decreases the number of phases and the area of the unaligned regions*. Λ_thres_* is the lowest coupling strength that gives rise to the complete alignment (two fully aligned phases). In the phase diagram presented in Figure 3a, this value is calculated for two cases of *N* = 5 and *N* = 10 at different χ values. The value of χ depends on the composition of the system. For larger χ values, larger coupling strength is required for complete alignment. Also, below a critical value for χ, χ*_crit_*, the multilayer does not phase separate and remains completely mixed. χ*_crit_* is close to 2 and increases slightly with the coupling parameter. Two curves χ*_crit_* and *Λ_thres_* divide the phase diagram into three regions of mix, multi-phase, and two-phase. For comparison *Λ_thres_* for N = 5 is also plotted (Figure 3a). Figure 3b shows that *Λ_thres_* increases with the degree of lamellarity. This is reasonable since the membrane multilayers with greater number of lamellae would require stronger interbilayer coupling strength to overcome the entropy.

In brief, Figure 3, clearly indicates that by increasing the number of lamellae, stronger coupling is required to align the domains, as expected. This is indeed in agreement with the experimental results presented in [1] that a multilayer with the number of lamellae more than ~1000 cannot be aligned under normal conditions.

Extending the model to a larger number of lamellae is computationally too expensive. Nevertheless, we expect similar phase diagram for larger number of lamellae once the threshold coupling parameter is accurately estimated.

It is important to mention that, prior to the recent experimental data reported in [1], the alignment of the domains in lipid multilayers was assumed to be impossible as entropy cannot trivially let formation of such ordering in the distribution of the domains. Moreover, previous reports of the alignment of the domains in multilayers with low number of lamellae (<5) attached to the substrates showed that the bilayers do not couple strongly to align the domains [22–24]. We believe that the reason of the unsuccessful alignment of the domains in the reported experiment is the influence of the substrate, which is most pronounced for the first few layers close to the substrate. Thus, we may conclude that although the coupling is strong, it cannot overcome the interaction between the substrate and the first few bilayers. Such an interaction is not taken into account in our model and the layers are assumed to be away from the substrate by at least a few layers.

The simulation was written and run using Matlab R2010a and Image analyses were performed using NIH Image J.

## 3. Conclusions

We presented a theoretical model which describes the phase separation in multicomponent lipid multilayers and the coupling involved in the alignment of the domains across several lamellae.

This work elucidates the phenomenon recently reported regarding the long-range interlayer alignment of the intra-layer domains in lipid multilayers [1]. The stacked lipid bilayers with such aligned domains have fundamental biological importance [10,11,25] with potentials for practical applications [13–15,26] which necessitate the detailed theoretical analysis of the subject.

We introduced an elemental phase diagram for multilayers with up to 10 bilayers. The value of the interlayer coupling for complete alignment was further calculated for the membranes with different intra-layer coupling parameters.

The surface area fraction of the multilayers occupied by different phases in different situations can be calculated with this model. To verify the model, experimental data were generated for a case study and the results were compared with those of the model calculations, which showed a good agreement.

## References

[1] Tayebi L., Ma Y., Vashaee D., Chen G., Sinha S., Parikh A. (2012). Long-range interlayer alignment of intralayer domains in stacked lipid bilayers. Nature Mater.

[2] Almeida P.F. (2009). Thermodynamics of lipid interactions in complex bilayers. Biochim. Biophys. Acta.

[3] Brown D.A., London E. (1998). Functions of lipid rafts in biological membranes. Annu. Rev. Cell Dev. Biol.

[4] Simons K., Ikonen E. (2000). Cell biology—How cells handle cholesterol. Science.

[5] Chazal N., Gerlier D. (2003). Virus entry, assembly, budding, and membrane rafts. Microbiol. Mol. Biol. Rev.

[6] Dietrich C., Bagatolli L.A., Volovyk Z.N., Thompson N.L., Levi M., Jacobson K., Gratton E. (2001). Lipid rafts reconstituted in model membranes. Biophys. J.

[7] Samsonov A.V., Mihalyov I., Cohen F.S. (2001). Characterization of cholesterol-sphingomyelin domains and their dynamics in bilayer membranes. Biophys. J.

[8] Veatch S.L., Keller S.L. (2002). Organization in lipid membranes containing cholesterol. Phys. Rev. Lett.

[9] Veatch S.L., Keller S.L. (2003). Separation of liquid phases in giant vesicles of ternary mixtures of phospholipids and cholesterol. Biophys. J.

[10] Bald D., Kruip J., Rogner M. (1996). Supramolecular architecture of cyanobacterial thylakoid membranes: How is the phycobilisome connected with the photosystems?. Photosynth. Res.

[11] Keegstra K., Cline K. (1999). Protein import and routing systems of chloroplasts. Plant Cell.

[12] Altamirano M. (1955). Electrical properties of the innervated membrane of the electroplax of electric eel. J. Cell. Comp. Physiol.

[13] Lenhert S., Brinkmann F., Laue T., Walheim S., Vannahme C., Klinkhammer S., Xu M., Sekula S., Mappes T., Schimmel T. (2010). Lipid multilayer gratings. Nat. Nanotechnol.

[14] Radler J.O., Koltover I., Salditt T., Safinya C.R. (1997). Structure of DNA-cationic liposome complexes: DNA intercalation in multilamellar membranes in distinct interhelical packing regimes. Science.

[15] Yamamoto J., Tanaka H. (2005). Dynamic control of the photonic smectic order of membranes. Nat. Mater.

[16] Frazier M.L., Wright J.R., Pokorny A., Almeida P.F. (2007). Investigation of domain formation in sphingomyelin/cholesterol/POPC mixtures by fluorescence resonance energy transfer and Monte Carlo simulations. Biophys. J.

[17] May S. (2009). Trans-monolayer coupling of fluid domains in lipid bilayers. Soft Matter.

[18] Safran S (1994). Statistical Thermodynamics of Surfaces, Interfaces, and Membranes.

[19] Davies H.T. (1996). Statistical Mechanics of Phases, Interfaces, and Thin Films.

[20] Pandit S.A., Jakobsson E., Scott H.L. (2004). Simulation of the early stages of nano-domain formation in mixed bilayers of sphingomyelin, cholesterol, and dioleylphosphatidylcholine. Biophys. J.

[21] Radhakrishnan A., Li X.M., Brown R.E., McConnell H.M. (2001). Stoichiometry of cholesterol-sphingomyelin condensed complexes in monolayers. Biochim. Biophys. Acta.

[22] Jensen M., Morris E., Simonsen A. (2007). Domain shapes, coarsening, and random patterns in ternary membranes. Langmuir.

[23] Simonsen A., Bagatolli L. (2004). Structure of spin-coated lipid films and domain formation in supported membranes formed by hydration. Langmuir.

[24] Pompeo G., Girasole M., Cricenti A., Cattaruzza F., Flamini A., Prosperi T., Generosi J., Castellano A. (2005). AFM characterization of solid-supported lipid multilayers prepared by spin-coating. Biochim. Biophys. Acta.

[25] Schmitz G., Muller G. (1991). Structure and function of lamellar bodies, lipid-protein complexes involved in storage and secretion of cellular lipids. J. Lipid Res.

[26] Xu J., Lavan D.A. (2008). Designing artificial cells to harness the biological ion concentration gradient. Nat. Nanotechnol.

